# Metastatic Lung Adenocarcinoma in a Lifetime Nonsmoker With an Atypical Radiological and Clinical Presentation

**DOI:** 10.7759/cureus.87943

**Published:** 2025-07-14

**Authors:** Yan Naing Tun, Fizza Mohsin, Muhammad H Khan, Shaurya Sharma, Ravikaran Patti

**Affiliations:** 1 Internal Medicine, Maimonides Medical Center, Brooklyn, USA; 2 Pulmonary and Critical Care Medicine, Maimonides Medical Center, Brooklyn, USA

**Keywords:** adenocarcinoma of the lung, atypical presentation, early detection of cancer, non-smoking, pulmonary nodule characterization

## Abstract

Lung cancer remains the leading cause of cancer-related deaths globally. While smoking-related lung cancers still account for most cases and cause approximately 100,000 deaths annually in the USA, smoking rates have been declining for decades. Lung cancer in never-smokers (LCINS), which tends to affect women and Asian populations, is now the fifth most common cause of cancer-related deaths worldwide. In 2023, over 20,000 lung cancer deaths in the USA were projected to occur in never-smokers, making LCINS the eighth leading cause of cancer-related mortality in the country. As the number of LCINS cases rises, it becomes increasingly important to explore the unique causes and characteristics of the disease, which calls for tailored diagnostic approaches and personalized treatment plans. Lung adenocarcinoma (LUAD) can present with atypical imaging features that often resemble benign conditions, including pneumonia, lung abscesses, post-infectious scarring, atelectasis, mediastinal masses, emphysema, and granulomatous disease. This variability in presentation can hinder accurate diagnosis and potentially delay timely treatment. While lung cancer is uncommon in younger individuals, with only 5.6% of new cases occurring in those under 54 years old, clinicians should maintain a high index of suspicion, as early detection is essential, and atypical cases can be easily missed. Here, we present a complex case involving an unusual radiologic manifestation of a lung mass, in which tuberculosis was initially considered the primary differential diagnosis.

## Introduction

Lung adenocarcinoma (LUAD) is the most common histologic subtype of lung cancer in the United States and globally, particularly among non-smoking populations [1-4]. While traditionally associated with tobacco exposure, an increasing number of cases are being identified in lifelong non-smokers, suggesting alternative risk factors, including genetic predisposition and environmental influences. In East Asia, approximately 30% of lung cancer cases occur in individuals with no history of smoking, compared to 10% in the United States [5-7]. While screening and therapeutic advancements have improved lung cancer care, late-stage diagnosis still accounts for more than 50% of cases, significantly impacting treatment options and outcomes [8].

Radiologically, LUAD can exhibit an extensive range of manifestations, often mimicking benign pulmonary conditions. Common imaging findings include spiculated nodules, ground-glass opacities, and lymphangitic spread. However, atypical presentations may resemble pneumonia, infectious abscesses, or post-inflammatory scarring, leading to diagnostic uncertainty and delays in treatment initiation [9].

Here, we report a case of a 43-year-old male Chinese migrant, a lifelong non-smoker, who presented with progressive dyspnea, significant weight loss, and nonspecific radiologic abnormalities initially suggestive of infectious pathology. Initial investigations raised concern for tuberculosis, delaying recognition of the underlying malignancy. However, further imaging and biopsy confirmed metastatic LUAD with lymphangitic spread and an epidermal growth factor receptor (EGFR) exon 19 deletion. The patient was subsequently initiated on osimertinib, a third-generation, irreversible EGFR tyrosine kinase inhibitor (TKI) specifically targeting EGFR mutations. This case highlights the importance of keeping a broad differential diagnosis for lung masses, especially in non-smokers presenting with atypical radiologic features.

This article was previously presented as a meeting abstract at the American Thoracic Society (ATS) International Conference in May 2024, held in San Diego, California.

## Case presentation

A 43-year-old East Asian male, a professional chef with no significant past medical history, a lifelong non-smoker, and no known family history of malignancy, presented to the emergency department with progressively worsening shortness of breath, non-productive cough, and fatigue over a three-month period. He reported an unintentional 30-pound weight loss over six months but denied fever, chills, night sweats, or hemoptysis. An outpatient chest X-ray revealed perihilar interstitial markings without evidence of infiltration.

**Figure 1:**
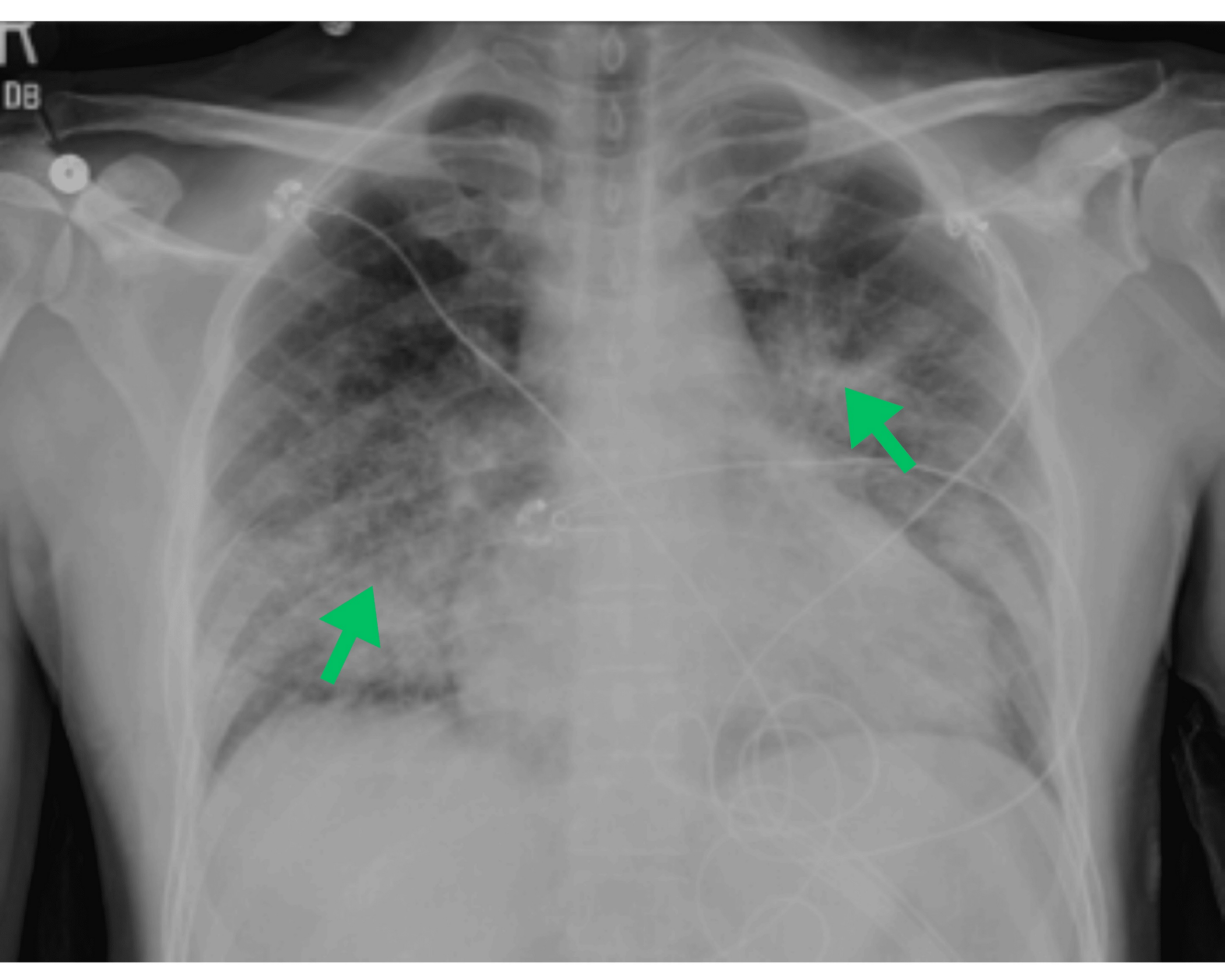
Initial chest X-ray demonstrating heterogeneous bilateral opacities with a reticulonodular pattern, along with perihilar interstitial markings and no signs of infiltration.

**Figure 2:**
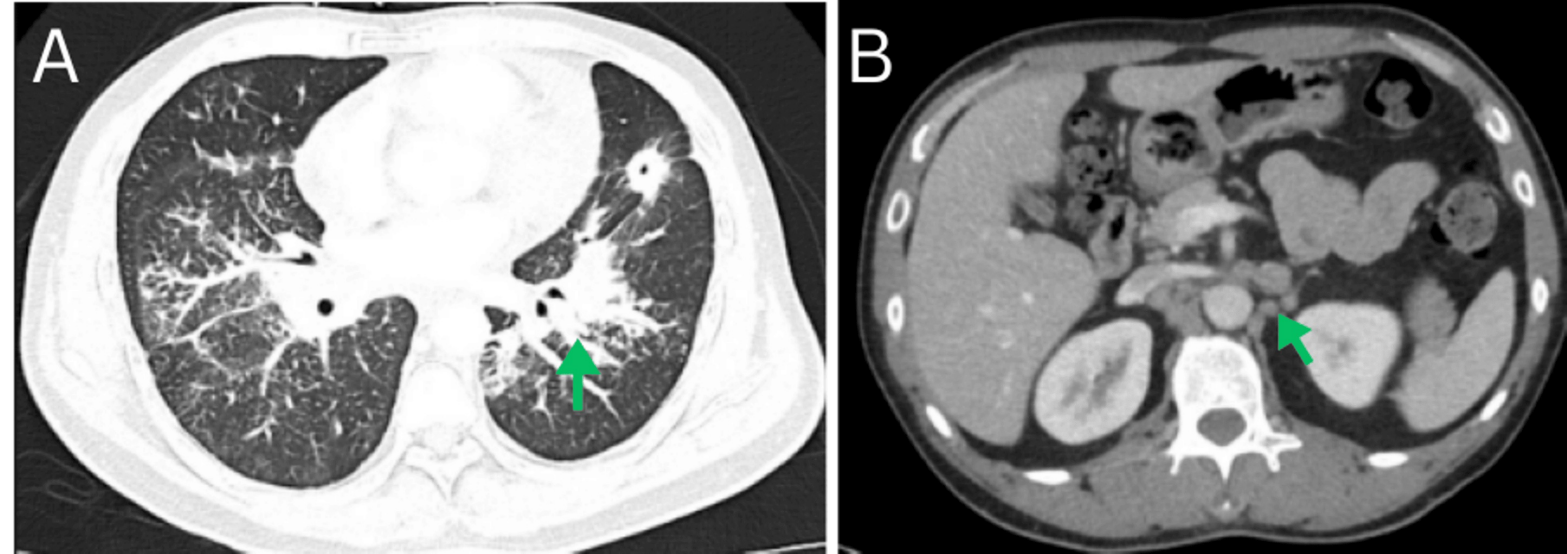
Admission chest (A) and abdominal (B) computed tomography (CT) axial sections. A: Admission chest computed tomography (CT) axial sections demonstrates a spiculated nodule with central cavitation in the left upper lobe. B: Abdominal CT reveals bilateral mediastinal and right hilar lymphadenopathy, raising suspicion for regional metastatic involvement or an underlying inflammatory condition.

**Figure 3:**
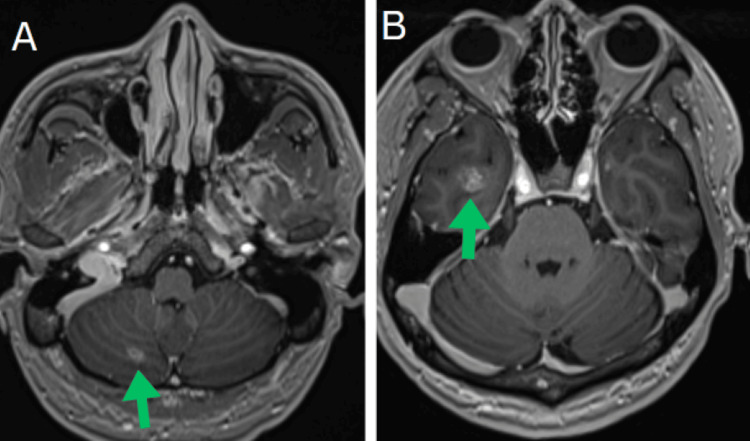
Initial brain magnetic resonance imaging (MRI) images. A: 7 mm enhancing lesion is present in the right inferomedial cerebellum. B: 1.1 cm enhancing lesion is seen superficially in the right inferior temporal lobe, accompanied by mild surrounding vasogenic edema and subtle petechial hemorrhage.

On arrival, the patient was tachycardic and tachypneic, requiring 3-4 L of supplemental oxygen via nasal cannula to maintain an oxygen saturation above 92%. Laboratory evaluation was notable for lactic acidosis of 3.2 mmol/L, pH of 7.47, PCO₂ of 29 mmHg, and PO₂ of 70 mmHg. A repeat chest X-ray in the emergency department revealed heterogeneous bilateral opacities with a reticulonodular pattern, as shown in Figure 1. Chest computed tomography (CT) revealed a 2.2 cm spiculated nodule with central cavitation in the left upper lobe, bilateral mediastinal and right hilar lymphadenopathy (up to 1.5 cm), and a small-volume pericardial effusion. Additional lymphadenopathy was noted in the gastrohepatic and retroperitoneal regions. Initial chest and abdominal CT findings are illustrated in Figure 2. Given the cavitary lesion and the patient’s origin from a high tuberculosis-endemic region, he was placed in airborne isolation for presumed Mycobacterium tuberculosis (MTB), although acid-fast bacteria (AFB) smears and cultures returned negative. Empiric antibiotics (ceftriaxone and doxycycline) were started for suspected superimposed pneumonia. Ceftriaxone was administered at 100 mg/kg (total dose: 1 g) every 24 hours, and doxycycline at approximately 7 mg/kg (total dose: 500 mg) per dose. As shown in Figure 3, the initial brain magnetic resonance imaging (MRI) revealed numerous intracranial enhancing lesions, most of which showed some surrounding vasogenic edema, favoring a metastatic etiology. Some of the lesions demonstrated trace hemorrhagic components.

**Figure 4:**
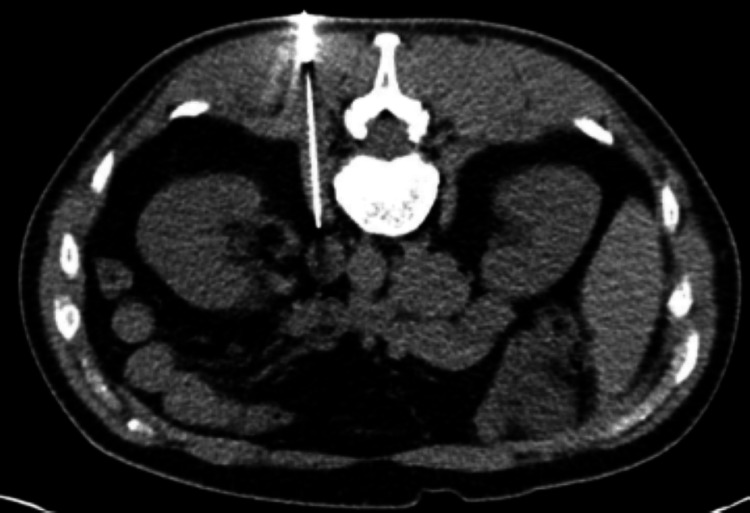
Computed tomography (CT)-guided core needle biopsy of a retroperitoneal lymph node. The figure demonstrates needle placement under real-time CT guidance for tissue sampling, which subsequently confirmed metastatic pulmonary adenocarcinoma.

As the patient’s clinical condition failed to improve, further imaging with CT of the abdomen and pelvis confirmed extensive retroperitoneal lymphadenopathy. A CT-guided biopsy of a left retroperitoneal lymph node performed by interventional radiology revealed metastatic pulmonary adenocarcinoma, as shown in Figure 4. Immunohistochemistry was positive for CKAE1/3, Cytokeratin 7 (CK7), thyroid transcription factor 1 (TTF-1), and Napsin A, supporting a diagnosis of lung adenocarcinoma. CK7 is typically expressed in the majority of lung adenocarcinomas (ADC). A lack of CK7 expression can complicate the diagnosis of pulmonary ADC, highlighting the importance of using a panel of immunomarkers, including TTF-1, Napsin A, p40, p63, and CK20, for accurate classification. Molecular analysis via liquid biopsy identified an EGFR exon 19 deletion. Targeted therapy with osimertinib (80 mg orally once daily) was initiated two weeks after diagnosis.

**Figure 5:**
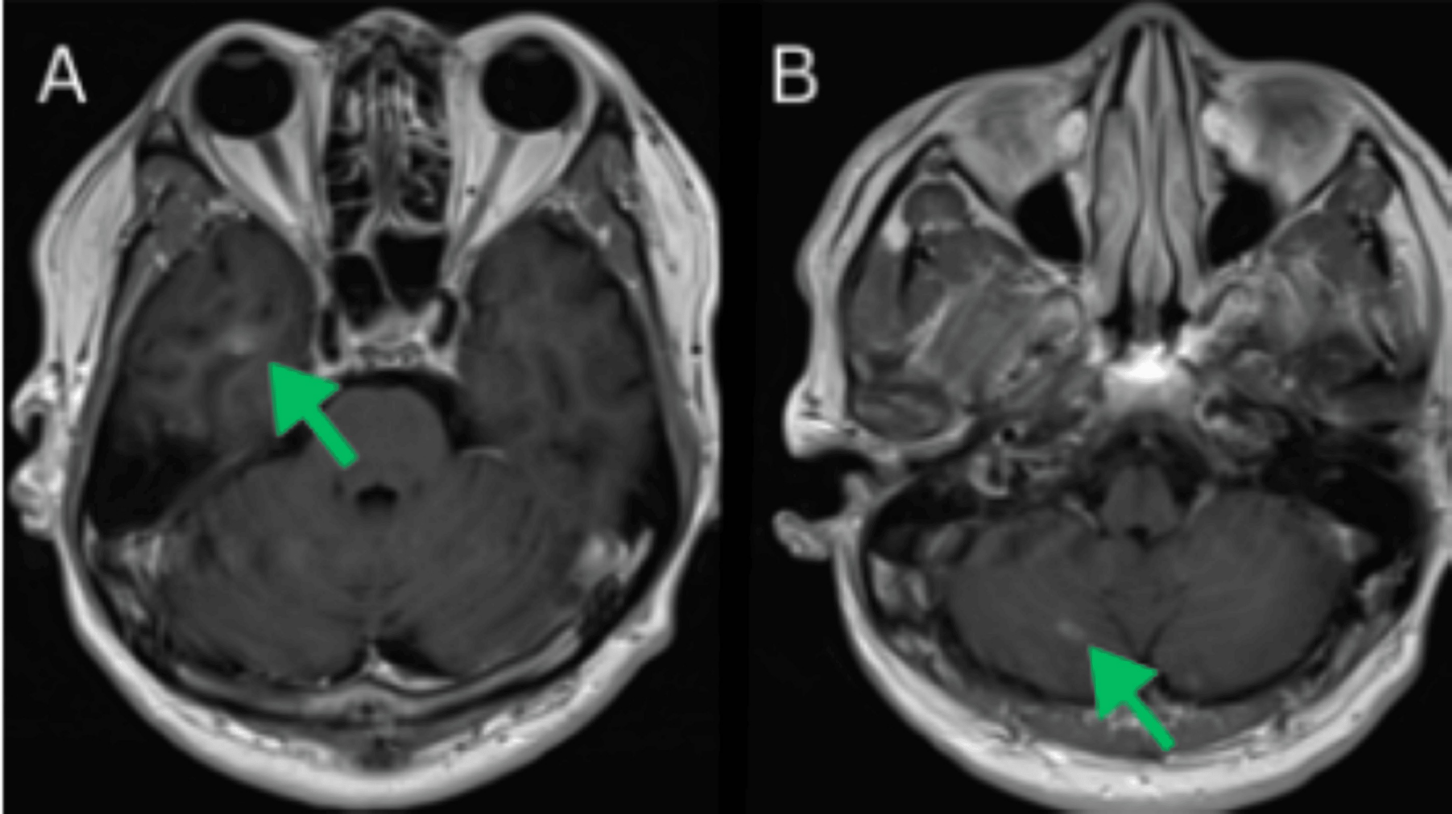
Brain magnetic resonance imaging (MRI) performed at the two-month follow-up A partial radiologic response was observed, with a reduction in the size and enhancement of the lesion in the right inferior temporal lobe (A) as well as the lesion in the right inferomedial cerebellum (B), indicating interval improvement in both regions.

**Figure 6:**
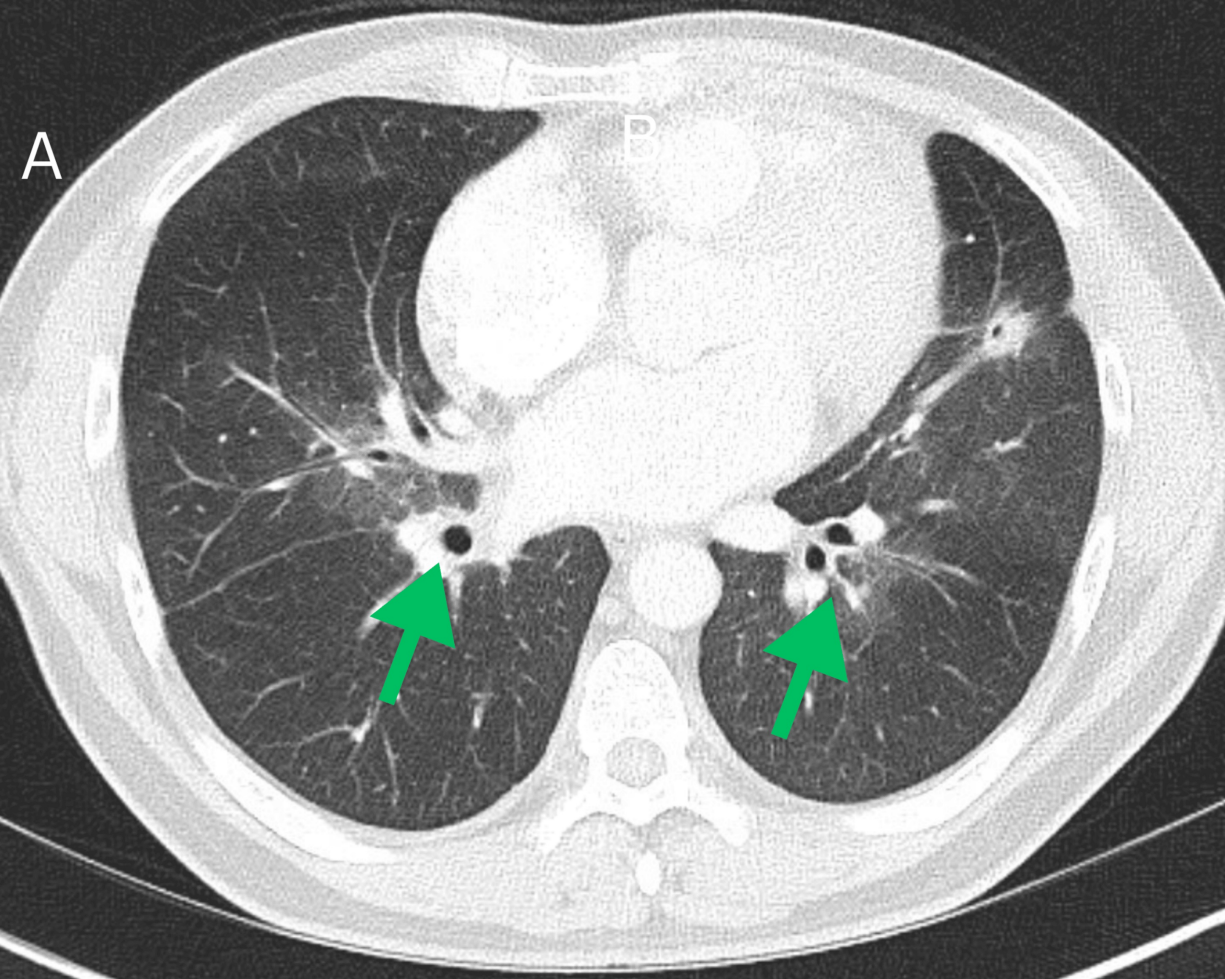
CT chest obtained at the two-month follow-up shows continued improvement in the primary tumor and lymphadenopathy, with an overall decrease in disease burden.

The patient reported symptomatic improvement, and a follow-up brain MRI at two months demonstrated a partial radiologic response, as shown in Figure 5. Correspondingly, the chest CT at the two-month follow-up, depicted in Figure 6, showed further reduction in the size of the primary lung lesion and associated lymphadenopathy, reflecting a continued decrease in overall disease burden.

Four months after diagnosis, systemic chemotherapy with carboplatin and pemetrexed was initiated, consisting of four cycles over 12 weeks. Cytotoxic chemotherapy was added to enhance disease control and potentially delay the emergence of resistance. He was then transitioned to maintenance pemetrexed while continuing osimertinib.

**Figure 7:**
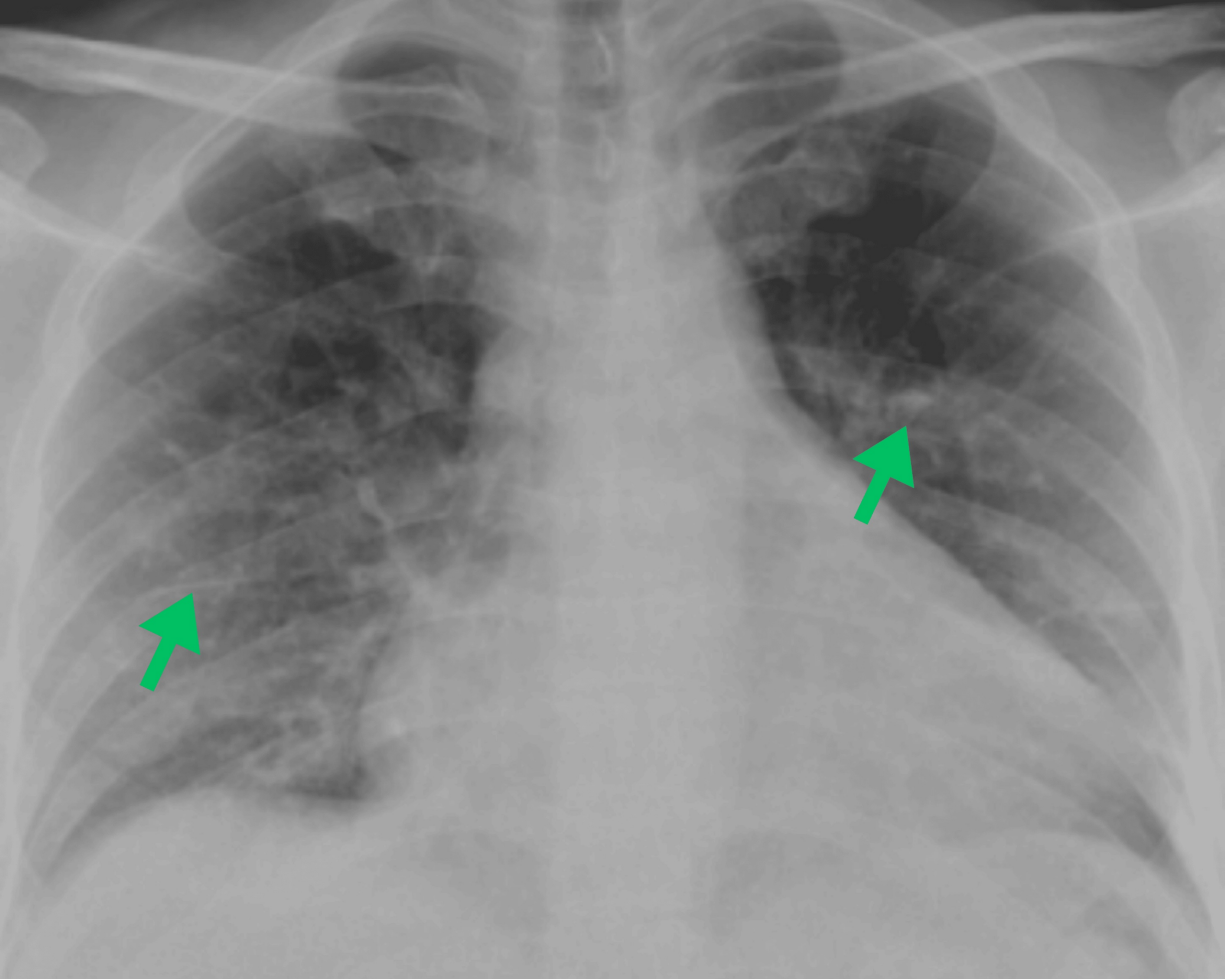
Follow-up chest radiograph at four months of treatment demonstrating marked improvement in bilateral pulmonary opacities and reduction of perihilar vascular congestion.

The patient was regularly followed during the treatment period, and a chest X-ray performed four months into therapy (Figure 7) revealed significant resolution of bilateral opacities and reduction in perihilar congestion.

**Figure 8:**
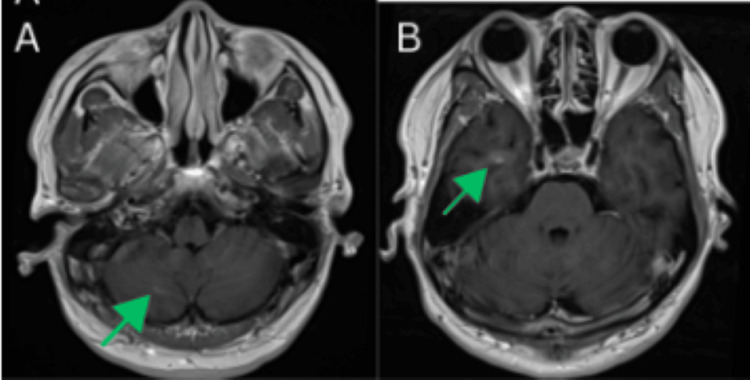
Brain magnetic resonance imaging (MRI) performed at the five-month follow-up. Follow-up magnetic resonance imaging (MRI) demonstrates small enhancing intra-axial lesions in the right inferomedial cerebellum (A) and the right inferior temporal lobe (B), both of which appear stable with no significant change in size, enhancement pattern, or surrounding edema compared to prior studies. These findings suggest no radiographic evidence of progression at this time.

**Figure 9:**
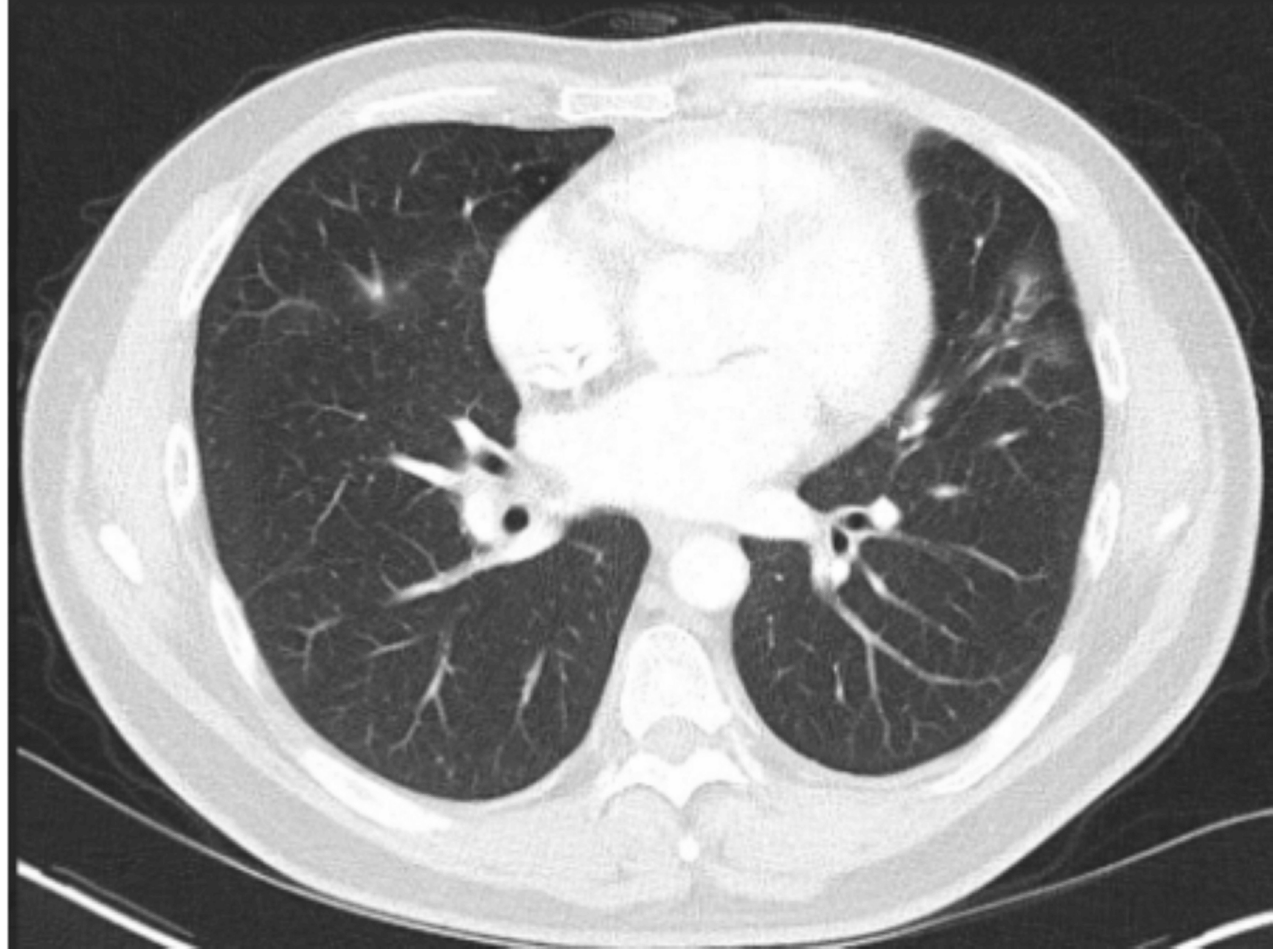
Chest computed tomography (CT) scan at the 18-month follow-up demonstrates marked improvement in the cavitary lesion and significant resolution of lymphadenopathy.

Subsequent CT chest imaging showed continued improvement in the primary tumor and lymphadenopathy. At five months after initiating chemotherapy, brain MRI revealed no new or enlarging enhancing lesions (Figure 8). The patient remained clinically stable and continued on maintenance therapy with osimertinib and pemetrexed. Follow-up chest CT imaging at 18 months (Figure 9) demonstrated a sustained radiologic response, with stable findings compared to previous scans and no evidence of new or progressive disease, indicating ongoing treatment effectiveness and disease control over the extended follow-up period. The patient has not required any surgical intervention or radiotherapy.

## Discussion

The case of our patient, a 43-year-old male Chinese immigrant and lifelong non-smoker, highlights important epidemiological, radiological, and molecular factors that shape both diagnosis and treatment strategies. His probable exposure to second-hand smoke, cooking fumes, and possible radon illustrates the complex and multifactorial nature of lung cancer development in non-smokers.

Lung cancer continues to be the leading cause of cancer-related mortality worldwide and among all ethnic groups in the United States. While most cases are associated with smoking, accounting for roughly 100,000 deaths each year in the U.S., a significant portion of lung cancers also occur in non-smokers. Approximately 10% of cases in the U.S. and nearly 30% in East Asia are diagnosed in individuals who have never smoked [5-7]. Lung cancer in non-smokers is linked to multiple risk factors, though none are definitively proven causes. Age increases risk, likely due to prolonged exposure to carcinogens like secondhand smoke and radon. Environmental exposures such as asbestos, air pollution, and cooking fumes also contribute. Genetic predisposition, including family history and mutations like EGFR, plays a key role. Underlying lung diseases and possible hormonal influences, especially estrogen, may further increase risk. While some viruses have been studied, evidence remains inconclusive. Overall, lung cancer in non-smokers results from a complex mix of genetic, environmental, and lifestyle factors [10]. In our patient's case, his occupation as a chef may have led to prolonged exposure to combustion-derived pollutants, which is a recognized risk factor. Furthermore, radon exposure, particularly common among individuals living in basement apartments, has been associated with increased lung cancer risk [10]. Notably, one study found that foreign-born Asian men and women had, on average, a 35% higher incidence of non-small cell lung cancer (NSCLC) compared to their U.S.-born counterparts [6].

When comparing survival rates between younger and older lung cancer patients matched for cancer stage, gender, and treatment, most large population-based studies and matched analyses show that younger patients experience equal or improved survival outcomes. This survival benefit is most evident in early-stage disease and is largely attributed to a lower burden of comorbidities and a greater likelihood of receiving aggressive treatment. For instance, younger individuals with NSCLC exhibit higher five-year survival rates at every stage compared to older adults, although this difference becomes less pronounced in advanced stages [11].

LUAD exhibits significant variability in CT imaging features, which can complicate early diagnosis [9]. In our patient, originally from a region with a high endemic prevalence of tuberculosis (TB), initial imaging revealed a cavitary lesion, leading to a differential diagnosis that favored tuberculosis. Due to the elevated TB burden in his country of origin, clinicians initially prioritized an infectious cause, which delayed consideration of malignancy. Cavitary lung lesions are commonly linked to infections such as TB or necrotizing pneumonia, often resulting in misdiagnosis in regions where these infections are prevalent. Typically, LUAD presents with ground-glass nodules; however, in this case, none were observed despite evidence of lymphangitic spread. Initial chest X-rays showed nonspecific perihilar interstitial markings, and follow-up imaging revealed opacifications, neither of which was strongly indicative of adenocarcinoma. This case highlights the diagnostic difficulty of atypical LUAD presentations, especially in patients from TB-endemic regions, where infectious etiologies are often the primary clinical consideration.

Due to the imaging similarities between LUAD and benign pulmonary conditions, a high level of clinical vigilance is essential. Clinicians should consider a wide differential diagnosis when evaluating respiratory symptoms alongside nonspecific imaging findings, especially in non-smoking patients with persistent and progressive symptoms [12]. 

Currently, there are no lung cancer screening guidelines for non-smokers. Symptoms are often vague and overlap with other diseases. Screening is generally recommended annually using low-dose computed tomography (LDCT) for high-risk individuals, particularly those aged 50 to 80 years with a 20 pack-year smoking history who are either current smokers or have quit within the past 15 years [13]. However, non-smokers with risk factors such as a family history should be closely monitored for symptoms that may warrant further testing.

Lung cancer differs significantly between smokers and non-smokers in both its pathological types and genetic makeup. Non-smokers and light or former smokers most commonly develop adenocarcinoma, while heavy smokers more frequently present with squamous cell carcinoma or small cell lung cancer [14]. At the molecular level, lung cancers in non-smokers often harbor specific genetic mutations such as those in the EGFR gene, KRAS mutations, and ALK rearrangements [15]. EGFR mutations are notably more prevalent in non-smokers, whereas Kirsten rat sarcoma viral oncogene homolog (KRAS) mutations occur in both groups but differ in their mutation types. KRAS mutations are the most common oncogenic alterations observed in patients with NSCLC. The KRAS plays a key role in the development of various solid tumors, including NSCLC. Among these mutations, the p.G12C single-nucleotide variant (KRAS^G12C) is the most frequently reported in NSCLC, occurring in approximately 12-13% of cases. For many years, KRAS mutations, including KRAS^G12C, were considered “undruggable” due to the lack of effective and well-tolerated targeted therapies. However, recent clinical trials - CodeBreaK100 [16] and KRYSTAL-1 [17] - have shown that sotorasib and adagrasib, two novel selective inhibitors of KRAS^G12C, demonstrate clinical efficacy with an acceptable adverse event profile in treating advanced NSCLC patients harboring this mutation. These variations suggest that lung cancer in non-smokers constitutes a distinct biological entity with unique molecular drivers.

The advent of targeted therapies has significantly changed the landscape of lung cancer treatment, particularly in cases with identifiable driver mutations. EGFR mutations, frequently found in non-smokers and individuals of East Asian descent, are associated with responsiveness to TKIs such as osimertinib. This drug specifically targets the T790M resistance mutation and has been shown to improve progression-free survival compared to standard chemotherapy [18]. As such, molecular profiling has become a cornerstone of modern lung cancer care, enabling precision treatments tailored to each patient’s genetic profile.

## Conclusions

Increasing awareness of the rare and atypical presentations of primary lung cancer, particularly lung adenocarcinoma in non-smokers, is essential to improving care for complex cases. Such cancers can mimic numerous benign pulmonary diseases on imaging, making early recognition and dedicated morphological assessment crucial for timely diagnosis. A high index of suspicion combined with early histopathological evaluation and molecular profiling enables the use of targeted therapies that significantly improve survival. Moreover, a multidisciplinary team approach enhances accurate diagnosis and guides personalized treatment, including the selection of the most appropriate and oncologically effective surgical procedures.

## References

[1] Li C, Lu H (2018). Adenosquamous carcinoma of the lung. Onco Targets Ther.

[2] Myers DJ, Wallen JM (2023). Lung adenocarcinoma. StatPearls [Internet].

[3] Lewis DR, Check DP, Caporaso NE, Travis WD, Devesa SS (2014). US lung cancer trends by histologic type. Cancer.

[4] Zhang Y, Vaccarella S, Morgan E (2023). Global variations in lung cancer incidence by histological subtype in 2020: a population-based study. Lancet Oncol.

[5] Subramanian J, Govindan R (2007). Lung cancer in never smokers: a review. J Clin Oncol.

[6] Raz DJ, Gomez SL, Chang ET (2008). Epidemiology of non-small cell lung cancer in Asian Americans: incidence patterns among six subgroups by nativity. J Thorac Oncol.

[7] Zhou W, Christiani DC (2011). East meets West: ethnic differences in epidemiology and clinical behaviors of lung cancer between East Asians and Caucasians. Chin J Cancer.

[8] (2025). SEER Cancer Statistics Review, lung and bronchus cancer. https://seer.cancer.gov/statfacts/html/lungb.html.

[9] Snoeckx A, Dendooven A, Carp L (2017). Wolf in sheep’s clothing: primary lung cancer mimicking benign entities. Lung Cancer.

[10] Laguna JC, Tagliamento M, Lambertini M, Hiznay J, Mezquita L (2024). Tackling non-small cell lung cancer in young adults: from risk factors and genetic susceptibility to lung cancer profile and outcomes. Am Soc Clin Oncol Educ Book.

[11] Arnold BN, Thomas DC, Rosen JE (2016). Lung cancer in the very young: treatment and survival in the National Cancer Data Base. J Thorac Oncol.

[12] Succony L, Rassl DM, Barker AP, McCaughan FM, Rintoul RC (2021). Adenocarcinoma spectrum lesions of the lung: detection, pathology and treatment strategies. Cancer Treat Rev.

[13] Dubin S, Griffin D (2020). Lung cancer in non-smokers. Mo Med.

[14] Travis LB, Gospodarowicz M, Curtis RE (2002). Lung cancer following chemotherapy and radiotherapy for Hodgkin's disease. J Natl Cancer Inst.

[15] Littman AJ, Thornquist MD, White E, Jackson LA, Goodman GE, Vaughan TL (2004). Prior lung disease and risk of lung cancer in a large prospective study. Cancer Causes Control.

[16] Skoulidis F, Li BT, Dy GK (2021). Sotorasib for lung cancers with KRAS p.G12C mutation. N Engl J Med.

[17] Jänne PA, Riely GJ, Gadgeel SM (2022). Adagrasib in non-small-cell lung cancer harboring a KRASG12C mutation. N Engl J Med.

[18] Hirashima T, Satouchi M, Hida T (2019). Osimertinib for Japanese patients with T790M-positive advanced non-small-cell lung cancer: a pooled subgroup analysis. Cancer Sci.

